# Conserved miR-10 family represses proliferation and induces apoptosis in ovarian granulosa cells

**DOI:** 10.1038/srep41304

**Published:** 2017-01-23

**Authors:** Tu Jiajie, Yang Yanzhou, Albert Cheung Hoi-Hung, Chen Zi-Jiang, Chan Wai-Yee

**Affiliations:** 1 Chinese University of Hong Kong – Shandong University Joint Laboratory for Reproductive Genetics, School of Biomedical Sciences, Faculty of Medicine, the Chinese University of Hong Kong, SAR, Hong Kong; 2 Key Laboratory of Fertility Preservation and Maintenance, Ministry of Education, Key Laboratory of Reproduction and Genetics in Ningxia, Department of Histology and Embryology, Ningxia Medical University, Yinchuan, Ningxia, 75004, P.R. China; 3 Center for Reproductive Medicine; National Research Center for Assisted Reproductive Technology and Reproductive Genetics; The Key Laboratory for Reproductive Endocrinology of Ministry of Education; Shandong Provincial Key Laboratory of Reproductive Medicine, Provincial Hospital Affiliated to Shandong University, Jinan, China

## Abstract

Granulosa cells (GCs) are essential somatic cells in the ovary and play an important role in folliculogenesis. Brain-derived neurotropic factor (BDNF) and the TGF-β pathway have been identified as a critical hormone and signalling pathway, respectively, in GCs. In this study, we found that a conserved microRNA family that includes miR-10a and miR-10b repressed proliferation and induced apoptosis in human, mouse, and rat GCs (hGCs, mGCs and rGCs, respectively). Moreover, essential hormones and growth factors in the follicle, such as FSH, FGF9 and some ligands in the TGF-β pathway (TGFβ1, Activin A, BMP4 and BMP15), inhibited miR-10a and miR-10b expression in GCs. In contrast, the miR-10 family suppressed many key genes in the TGF-β pathway, suggesting a negative feedback loop between the miR-10 family and the TGF-β pathway in GCs. By using bioinformatics approaches, RNA-seq, qPCR, FISH, immunofluorescence, Western blot and luciferase reporter assays, BDNF was identified as a direct target of the miR-10 family in GCs. Additionally, reintroduction of BDNF rescued the effects of miR-10a and miR-10b in GCs. Collectively, miR-10a and miR-10b repressed GC development during folliculogenesis by repressing BDNF and the TGF-β pathway. These effects by the miR-10 family on GCs are conserved among different species.

Follicular development can be divided into several stages, including primordial, primary, secondary, and tertiary follicles^1^. It is well-known that follicles are crucial factors in the ovary, with each follicle consisting of an oocyte surrounded by granulosa cells (GCs). Oocytes gradually increase in size and progress to maturation accompanied by proliferation and differentiation of the surrounding somatic cells, including GCs, theca cells (TCs) and other somatic cells^2^. The proliferation and differentiation of GCs are key events during follicle development. This process is regulated by complex communication among oocytes, GCs, TCs and other somatic cells. Brain-derived neurotropic factor (BDNF) has been shown to play a direct role in regulating early follicular development and ovulation in several mammalian species^3^,^4^. It also plays an autocrine role in cell proliferation and survival in granulosa cells^5^. Additionally, certain extra- and intra-ovarian hormones and pathways, such as follicle-stimulating hormone (FSH), fibroblast growth factor 9 (FGF-9) and the TGF-β superfamily, have been found to be involved in regulating follicular development and oocyte maturation^6^.

microRNAs (miRNAs) are single-stranded, 19–25 nucleotide non-coding RNAs that bind to target mRNAs and mediate translational repression and/or mRNA degradation^7^. miRNAs control many vital biological processes, including ovarian function^8^. microRNAs have also been shown to be instrumental for defining specific effects in post-natal miRNA deficiency, such as those involved in female fertility and folliculogenesis^9^,^10^. Taken together, these data suggest that aberrant miRNA expression is closely related ovary and female reproductive tract diseases^11^.

miR-10 family members are encoded in evolutionarily conserved loci within the Homeobox (*Hox*) gene clusters^12^. Co-expression of *miR-10* and *Hox* genes during development^13^ and experimental evidence of miR-10 targeting of *HOX* transcripts^14^ have suggested a role for this miRNA family in development. Mammalian *miR-10a* and *miR-10b* are located upstream of *HoxB4* and *HoxD4,* respectively. They exhibit a high degree of sequence conservation, differing at only the eleventh nucleotide (U and A, respectively), which thermodynamically enables them to target fully overlapping mRNAs^15^. It is well-known that the *Hox* gene clusters play important roles in the development of the reproductive system^16^. The miR-10 family has been found to include specific microRNA markers in normal granulosa cells from the ovary^17^,^18^. Therefore, a number of questions regarding the function of the miR-10 family in ovarian granulosa cells need to be addressed.

In the present study, we demonstrated that both miR-10a and miR-10b suppressed human, mouse and rat granulosa cell proliferation by targeting BDNF, while reintroduction of BDNF blocked the inhibitory effects of miR-10a and miR-10b on GC proliferation and apoptosis. The miR-10 family was also regulated by a number of key hormones and growth factors in the ovary and formed a negative feedback loop with the TGF-β pathway in GCs. Taken together, this study revealed that the miR-10 cluster might play an important role in ovarian folliculogenesis by repressing GC development.

## Results

### miR-10 family is highly conserved among different species

**Figure 1:**
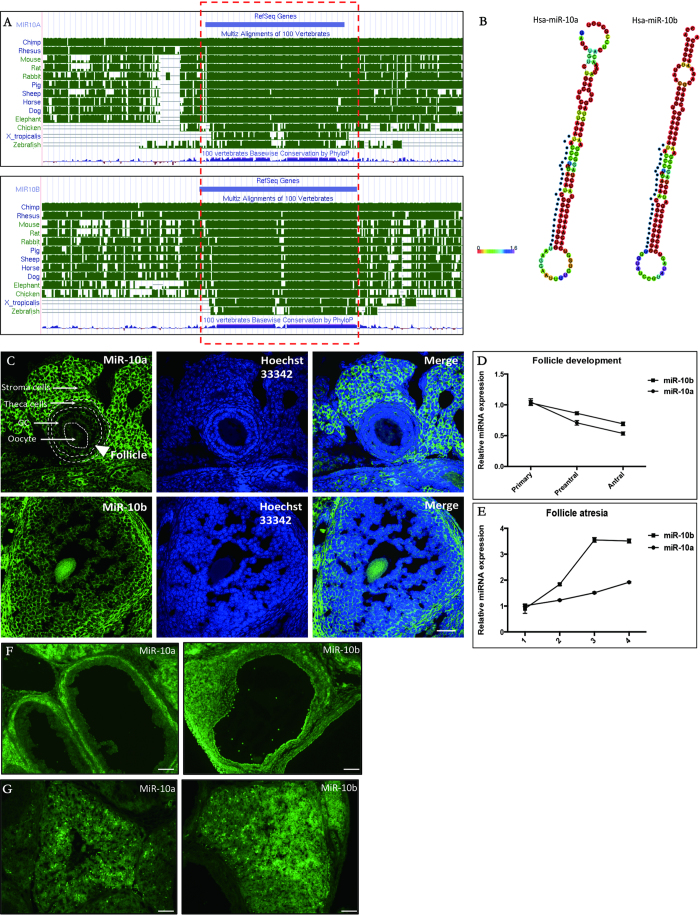
miR-10 family expression in normal and atretic granulosa cells. (**A**) Mature miR-10a and miR-10b sequences are highly conserved among different species. (**B**) Stem-loop structures for human pre-miR-10a and miR-10b predicted by the Vienna RNAfold (mature miRNA sequences are indicated by dots). (**C**) FISH for miR-10a and miR-10b in mouse ovary. (**D** and **E**) Expression patterns for miR-10a and miR-10b during follicle development and follicle atresia. (**F** and **G**) FISH for miR-10a and miR-10b in atretic follicles.

miR-10 was identified as a specific marker for mouse granulosa cells from previous miRNA-sequencing results^17^. It was also reported that miR-10 could repress proliferation in porcine granulosa cells^19^. However, the function of the miR-10 family is still unknown in other species, such as humans, mice and rats. Here, we identified and characterized the genes encoding human miR-10a and miR-10b, two members of the miR-10 family, in human, mouse and rat GCs. The nucleotide sequence of the miR-10a and miR-10b precursors are highly conserved in mammals (Fig. 1A). The mature hsa-miR-10a-5p and hsa-miR-10b-5p sequences are UACCCUGUAGAUCCGAAUUUGUG and UACCCUGUAGAACCGAAUUUGUG, respectively, and have only one different nucleotide. The mature miR-10a and miR-10b sequences are also quite conserved among human, mouse and rat; miR-10a is identical among human, mouse and rat, while there is also only one nucleotide change in rno-miR-10b compared to human and mouse miR-10b (U to C at nucleotide 11) (Supplementary Fig. 1A). The seed region (UCAAGUA) of miR-10 is conserved among vertebrate species. We also identified six asymmetric bulges in the structures of the hsa-miR-10a and hsa-miR-10b duplexes (Fig. 1B). These results indicate that the miR-10a and miR-10b precursors and mature sequences are highly conserved and might have similar functions in mammals.

### Identification of miR-10a and miR-10b in granulosa cells

Using fluorescence *in situ* hybridization (FISH), both miR-10a and miR-10b were shown to be expressed in mouse and rat GCs (Fig. 1C and Supplementary Fig. 1C). However, their expression in granulosa cells was much lower than in theca cells in follicles and stroma cells outside the follicle. Both miR-10a and miR-10b gradually decreased during follicle maturation (Fig. 1D) and increased by follicle atresia, as determined by RT-qPCR (Fig. 1E). miR-10 family expression was abundant in the remaining GCs in atretic follicles (Fig. 1F). These microRNAs were also highly expressed in the interstitial gland, which is differentiated from atretic secondary follicles (Fig. 1G). Taken together, these results suggest that miR-10a and miR-10b might play a negative role in follicle development.

### miR-10a and miR-10b repress proliferation and induce apoptosis in human, mouse and rat granulosa cells

**Figure 2:**
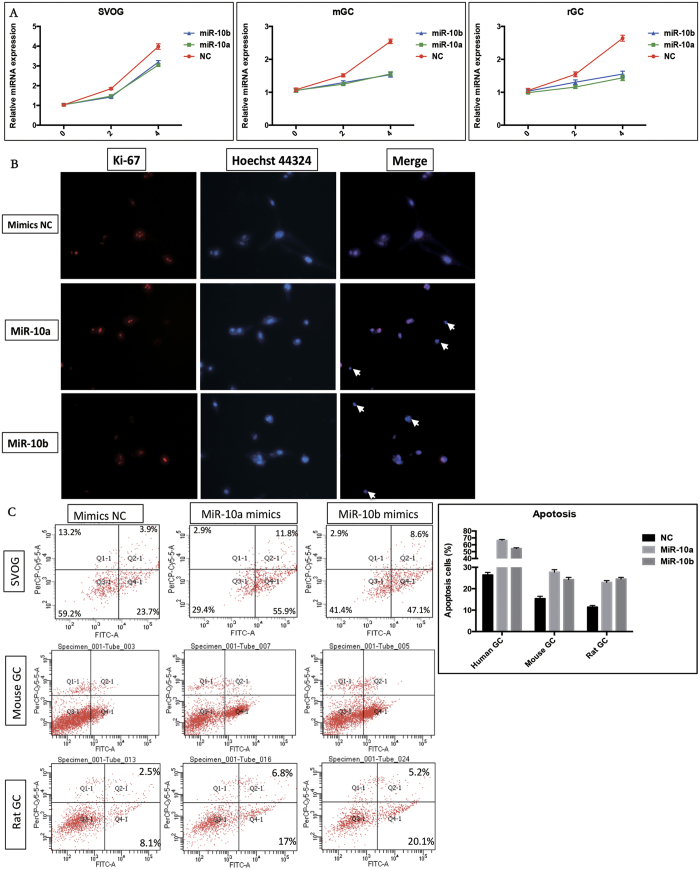
miR-10 family members repressed proliferation and induced apoptosis in granulosa cells. (**A**) miR-10a and miR-10b repressed proliferation in human, mouse and rat GCs. (**B**) miR-10a and miR-10b repressed human GC cell proliferation by Ki-67 staining. (**C**) miR-10a and miR-10b induced apoptosis in human, mouse and rat GCs.

To further investigate the role of the miR-10 family in GCs, both miR-10a and miR-10b were induced in GCs by transfection with miRNA mimics (Supplementary Fig. 1B). miR-10a and miR-10b mimic treatment significantly reduced the viability of human, mouse and rat GCs (Fig. 2A). By using Ki-67 staining, the proliferation of GCs was also found to be repressed by the miR-10 family (Fig. 2B). In contrast, apoptosis was induced by miR-10a and miR-10b in GCs of different species (Fig. 2C). These data confirmed that the miR-10 family simultaneously represses proliferation and induces apoptosis in GCs; this effect is conserved among humans, mice and rats.

### miR-10a and miR-10b expression in granulosa cells is regulated by extrinsic/intrinsic signals

**Figure 3:**
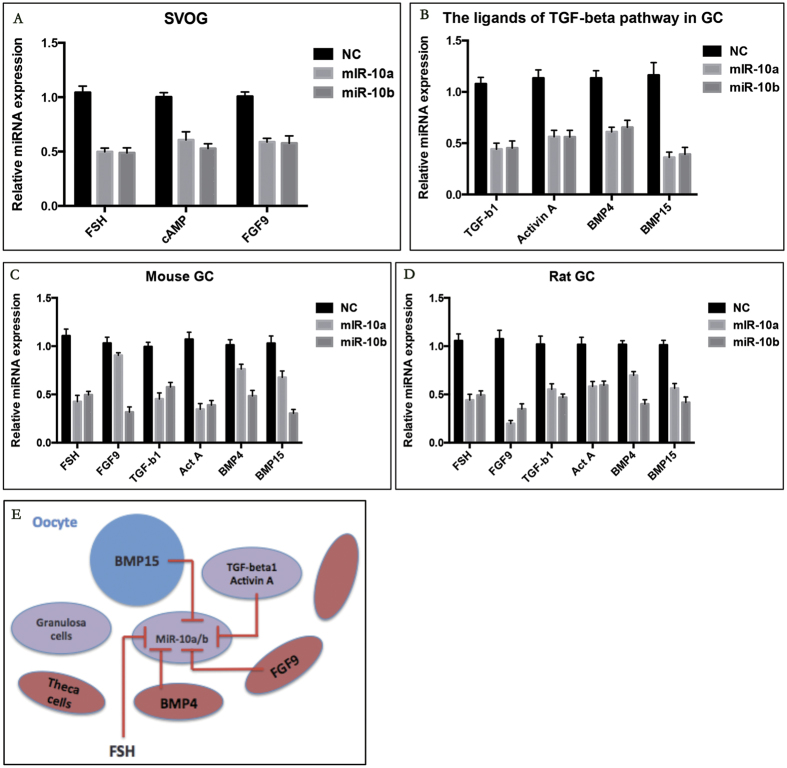
Effects of exposure to hormone and growth factors on miR-10a and miR-10b in granulosa cells. (**A**) Effects of exposure to FSH and FGF9 on miR-10a/b in human GCs. (**B**) Effects of exposure to TGF-β superfamily ligands on miR-10a/b in human GCs. (**C** and **D**) Effects of exposure to hormones and growth factors on miR-10a/b in mGCs and rGCs. (**E**) A schematic summary of the interactions between hormones/growth factors and the miR-10 family within follicles.

Previous studies have shown that both extrinsic and intrinsic signals play important roles during follicle development. Follicle-stimulating hormone (FSH) could stimulate granulosa cells to convert androgens to oestradiol via aromatase^20^ and maintain GC proliferation and maturation^21^. miR-10a and miR-10b were significantly decreased by recombinant human FSH in hGCs, mGCs and rGCs (Fig. 3A). As expected, the mediator of FSH in GCs, cAMP, also greatly repressed miR-10a and miR-10b in GCs (Fig. 3A). Fibroblast growth factor 9 (FGF9) which is secreted by theca cells, was another important local paracrine regulator of follicle function^22^. The results showed that FGF9 could also greatly repress the miR-10 family in GCs (Fig. 3A). Follicle development and function are modulated by the spatial and tissue-specific expression of TGF-β superfamily members^23^. Here, RT-qPCR analysis was used to measure changes in the abundance of miR-10a and miR-10b in a cultured human GC line and mouse and rat primary GCs following exposure to TGF-β superfamily ligands. Human GCs were treated with vehicle control or recombinant BMP4, BMP7 (theca cell-derived), BMP15 (oocyte-derived), Activin A (granulosa cells-derived) or TGF-β1. As shown in Fig. 3B, treatment with these TGF-β superfamily ligands inhibited miR-10a and miR-10b in GCs. Similar results were also obtained with FSH-, TGF-β1- and Act A-treated mGCs and rGCs (Fig. 3C).

To summarize, these results showed that FSH, FGF9 and TGF pathway signalling could inhibit miR-10a and miR-10b expression in hGCs, mGCs and rGCs, which suggests that the FSH/FGF9 and TGF-β pathway may function as an upstream regulator of miR-10a and miR-10b in GCs; these effects were conserved among different species (Fig. 3D).

### The general function of the miR-10 family in granulosa cells

**Figure 4:**
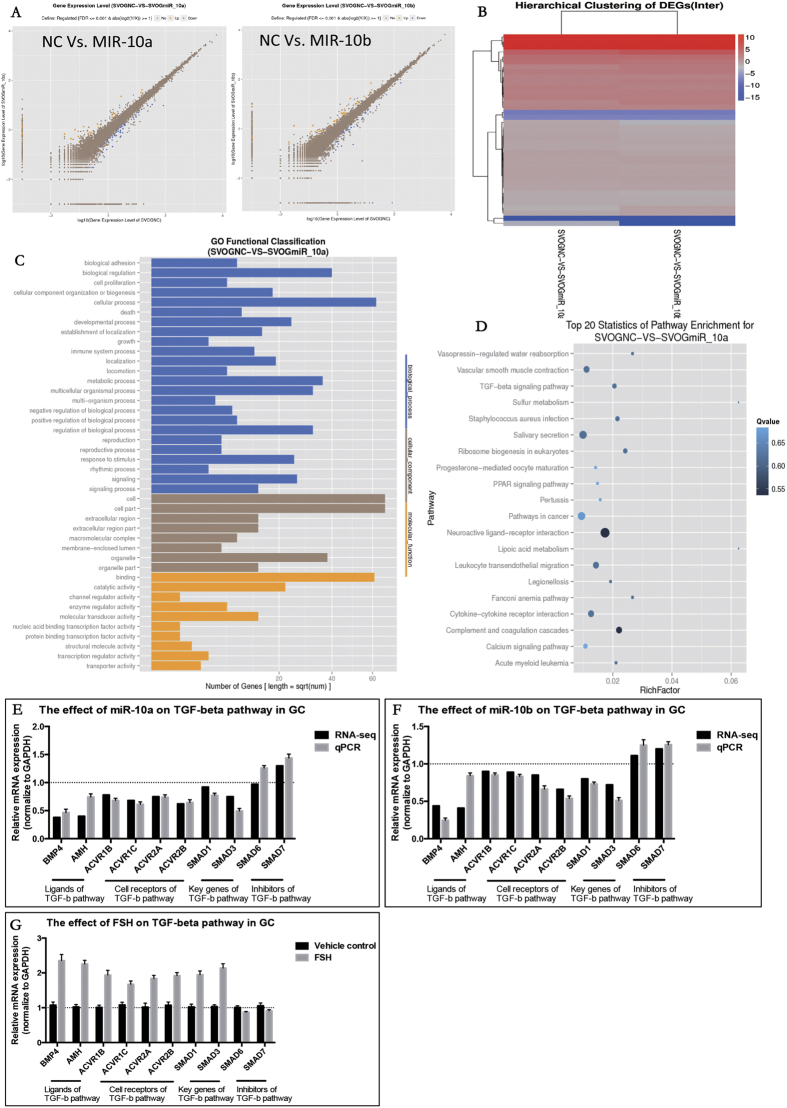
The effect of the miR-10 family on GCs on a transcriptome-wide scale. (**A**) Scatter chart of all the expressed genes in GCs overexpressing miR-10a and miR-10b by RNA-seq. (**B**) Hierarchical clustering of the DEGs in GCs overexpressing miR-10a and miR-10b. (**C**) Gene Ontology analysis in GCs overexpressing miR-10a and miR-10b. (**D**) Pathway analysis in GCs overexpressing miR-10a and miR-10b (**E**) The effects of miR-10a/b on key factors in the TGF-β pathway.

To further identify the associated pathways and direct targets of miR-10a and miR-10b in GCs, RNA-seq was performed for miR-10a/b-overexpressing granulosa cells (Fig. 4A). Hierarchical clustering of the differentiated expressed genes (DEGs) showed that the DEG expression patterns were quite similar regardless of whether miR-10a or miR-10b was overexpressed in GCs, implying similar functions for miR-10a and miR-10b in GCs (Fig. 4B). Gene ontology results suggested that miR-10a and miR-10b target genes were highly related to cell growth, proliferation, development and reproduction (Fig. 4C and Supplementary Fig. 1D). Additionally, signalling pathway analysis showed that the TGF-β pathway was a potential candidate pathway (Fig. 4D and Supplementary Fig. 1E). Some essential genes in this pathway, including ACVR2A, ACVR2B, SMAD1, SMAD3, BMP4 and AMH, were significantly repressed by both miR-10a and miR-10b in GCs (Fig. 4E). Considering that TGF-β superfamily ligands could greatly repress the miR-10 family in GCs, this result suggests that the miR-10 family and the TGF-β pathway might be involved in a negative feedback loop.

### BDNF is a direct target of the miR-10 family in granulosa cells

**Figure 5:**
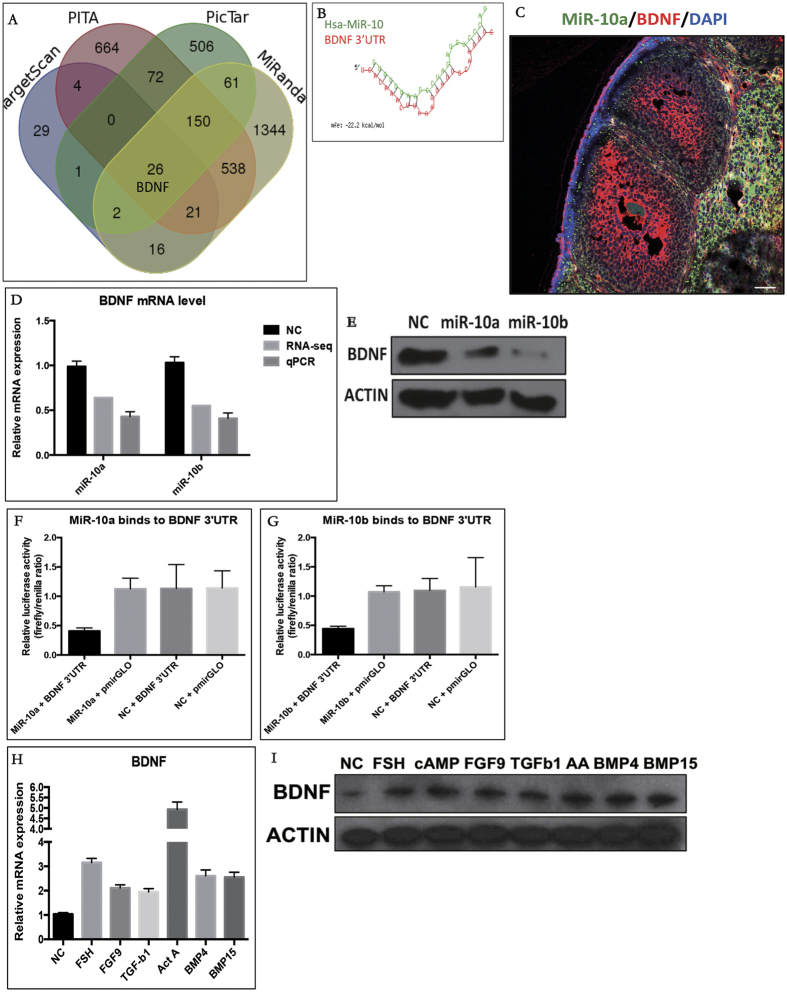
BDNF is a direct target of miR-10a/b in GCs. (**A**) Predicted targets of miR-10 family from four algorithms. (**B**) The miR-10 binding site within the BDNF 3′-UTR was predicted by RNAhybrid. (**C**) BDNF expression was opposite to that of miR-10a/b in GCs. (**D**) The effects of the miR-10 family on BDNF mRNA levels in GCs. (**E**) The effects of the miR-10 family on BDNF protein levels in GCs. (**F**) The luciferase reporter assay showed direct binding between the miR-10 family and the BDNF 3′ UTR.

By combining the RNA-seq and miRNA target prediction software results, BDNF was predicted to be a potential direct target of miR-10a/b in GCs (Fig. 5A). A putative miR-10 binding site in the BDNF 3′-untranslated region (UTR) was also identified (Fig. 5B). The opposite expression patterns for BDNF and the miR-10 family in GCs further indicated the repressive effect of miR-10a/b on BDNF (Fig. 5C). As validated by RT-qPCR, BDNF was inhibited at the mRNA by the miR-10 family in GCs (Fig. 5D). As shown in Fig. 5E, miR-10a/b overexpression led to a significant decrease in BDNF protein levels compared with the negative control. To determine whether BDNF was direct target of miR-10a and miR-10b, the putative miR-10 target 3′ UTR was cloned into a reporter plasmid downstream of a luciferase gene (Fig. 5F). The results showed that both the miR-10a and miR-10b mimics repressed the fluorescence from the 3′ UTR compared with the negative control, indicating that miR-10a and miR-10b could directly bind to the BDNF 3′ UTR. These results indicated that BDNF was a direct target of miR-10a and miR-10b in GCs.

### BDNF rescues miR-10a- and miR-10b-induced proliferation repression and apoptosis induction in GCs

**Figure 6:**
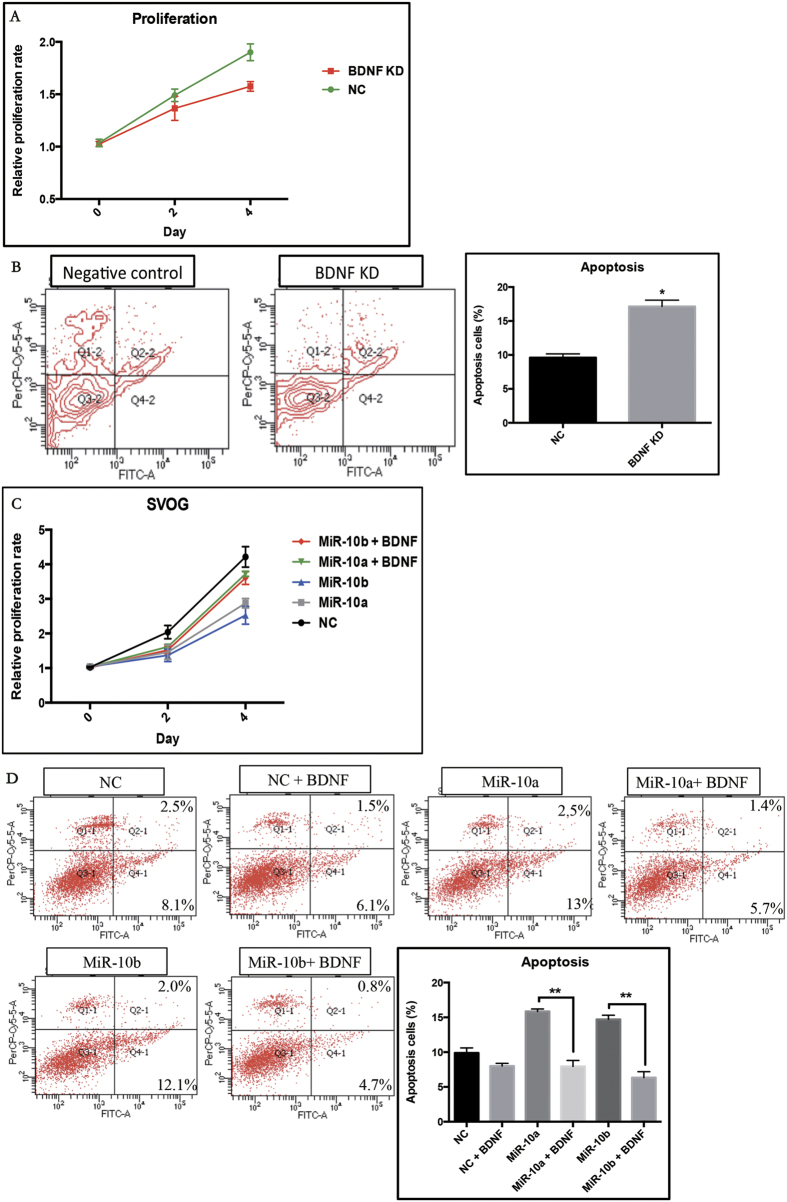
BDNF rescues miR-10 family-caused effects in GCs. (**A**) Proliferation was inhibited by BDNF knockdown in GC. (**B**) Apoptosis was induced by BDNF knockdown in GCs. (**C**) BDNF rescued miR-10a/b-induced repression of proliferation in GCs. (**D**) BDNF rescued miR-10a/b-induced apoptosis in GCs. Each bar in the figure represents the mean ± SEM from eight replicates. *P < 0.05, **P < 0.01 and ***P < 0.0001.

To prove that the downstream target of the miR-10 family, BDNF, could mediate the function of the miR-10 family in GCs, shRNA was used to knockdown BDNF in GCs (Supplementary Fig. 1G). Similar to GCs overexpressing the miR-10 family, proliferation was inhibited (Fig. 6A) and apoptosis was induced (Fig. 6B) upon BDNF knockdown. To further confirm that the anti-proliferative and pro-apoptotic functions of the miR-10 family in GCs are at least partially via BDNF, recombinant BDNF was used to treat miR-10a/b-overexpressing GCs. The reintroduction of BDNF into GCs promoted proliferation compared with the negative control, indicating that BDNF had a positive effect on GC proliferation (Fig. 6C). To further explore whether BDNF mediates the function of the miR-10 family in GC apoptosis, GCs were co-treated with miR-10 family mimics in the presence of recombinant BDNF or vehicle control. As expected, BDNF could rescue GC apoptosis caused by miR-10a and miR-10b mimic transfection (Fig. 6D). Taken together, the results showed that BDNF could at least partially mediate the function of the miR-10 family in GCs.

## Discussion

Normal proliferation and differentiation of granulosa cells are critical for the development of ovarian follicles, as abnormal growth of granulosa cells leads to infertility^24^. Despite the elucidated role of many genes in promoting the proliferation of granulosa cells and the growth of preantral follicles^25^, little is known regarding post-transcriptional regulation mechanisms for these genes. Here, we demonstrated that two members in the miR-10 family, miR-10a and miR-10b, function as anti-proliferation and pro-apoptosis factors in human, mouse and rat GCs by directly targeting the 3′ UTR of BDNF in GCs. We also identified that miR-10a/b and the TGF-β pathway form a negative feedback loop in granulosa cells.

MiRNAs are small non-coding RNAs that repress mRNA translation at the post-transcriptional level^7^. Many miRNAs that share a common “seed sequence” form a family and are located on different chromosomes in the genome; one such example is the miR-10 family, which is a highly conserved family among different species. The miR-10 family has two members, miR-10a and miR-10b. Each of these miRNAs is embedded within intragenic regions and might share a common promoter of two genes in the HOX cluster, HOXB4 and HOXD4. Based on small RNA-seq from a previous study, miR-10 is a specific marker for mouse granulosa cells^17^. Therefore, we characterized the miR-10 family and found that the functions of both miR-10a and miR-10b are highly conserved in humans, mice and rats. These results indicate that the miR-10 family has similar functions in GCs in different species.

The important roles of several hormones or growth factors, such as BMP15^26^ from oocytes, Activin A from granulosa cells^27^, BMP4/FGF9 from theca cells^22^,^28^ and even FSH^29^ from outside follicles during folliculogenesis, have been proven. In this study, we found that these critical regulatory factors could repress miR-10a and miR-10b in granulosa cells, further indicating the negative roles of the miR-10 family during folliculogenesis. BMP4 and BMP15 are from the BMP family, and Activin A is a member of the Activin family, and all are components of the TGF-β pathway^23^, suggesting that the TGF-β signalling pathway might also repress the miR-10 family in GCs. Therefore, we tested the effect of recombinant TGF-β1 on miR-10a/b in GCs. Both miR-10a and miR-10b were induced by TGF-β1 in GC. By using RNA-seq and qPCR, miR-10a and miR-10b were shown to inhibit many key genes within the TGF-β pathway, including ligands, receptors and transcription factors. These results indicate that autocrine and/or endocrine signals from hormones or growth factors during granulosa cell differentiation are involved in repressing the miR-10 family in GCs. Additionally, the TGFβ pathway and miR-10a/b form a negative feedback loop in GCs.

By using RNA-seq screening, bioinformatics prediction, qPCR, Western blot analysis, luciferase reporter assays and FISH-IF validation, BDNF was identified as a direct target of the miR-10 family in GCs. As a neurotrophic growth factor, BDNF has been reported to play both autocrine and paracrine roles in ovary development, such as promoting oocyte development and maturation^30^,^31^. Consistent with these observations, our data showed that the miR-10 family decreased proliferation and induced apoptosis in granulosa cell. miR-10a and miR-10b directly targeted BDNF in GCs, suggesting that the miR-10 family might also affect other normal ovary functions apart from GC development. Thus, it is definitely worth further attention and study.

In conclusion, we found that miR-10 family members, including miR-10a and miR-10b, are expressed at basal levels in GCs but are highly expressed in theca and stroma cells within the ovary. Both miR-10a and miR-10b could repress proliferation and induce apoptosis in human, mouse and rat granulosa cells, at least partly through repressing BDNF by directly binding to its 3′ UTR. Additionally, many hormones and growth factors in the ovary repressed the miR-10 family in GCs. Moreover, the miR-10 family and the TGF-β pathway form a negative feedback loop in GCs. This study provides new insights into how the miR-10 family functions in the female reproductive system. Manipulation of this miRNA family and its targets may have implications in the treatment of female reproductive diseases, such as polycystic ovarian syndrome (PCOS).

## Materials and Methods

### Animals

The 23–26-day-old C57 mice and S/D rats were purchased from the Laboratory Animal Services Centre (LASEC) at the Chinese University of Hong Kong (CUHK, Hong Kong, China) and maintained in the Animal Holding Core in the Lo Kwee-Seong Integrated Biomedical Sciences Building, CUHK. All of the animal experiments were approved by the Department of Health, Hong Kong. We can confirm that all of the methods were performed in accordance with the relevant guidelines and regulations.

### Cell lines

The human granulosa cell line (SVOG) was obtained from Shandong University and cultured in DMEM/F12 (Gibco BRL/Invitrogen, Carlsbad, CA, USA) supplemented with 10% foetal bovine serum and 1% penicillin–streptomycin. HEK293T cells were kept in our lab and cultured in DMEM supplemented with 10% foetal bovine serum and 1% penicillin–streptomycin. All of the cells were cultured in a humidified atmosphere (5% CO_2_) at 37 °C. Cultured cells were dislodged from the culture flask using a trypsin (0.25%)/EDTA solution when passaged.

### Granulosa cell isolation and culture

mGCs and rGCs were collected from the ovaries of 23–26-day-old mice and rats, respectively, using the follicular puncture method as previously described^32^. Briefly, C57 mice and S/D rats were superovulated by intraperitoneal injection of PMSG (Pregnant Mare Serum Gonadotropin, Sansheng Pharmaceutical Co., Ltd., Ningbo, China). After approximately 48 hours, the animals were euthanized by cervical dislocation, and the ovaries were collected into PBS through an abdominal incision under aseptic conditions. Then the ovarian follicles were punctured by needle (1 ml injection syringe) under a stereoscopic microscope. The released cells were scattered and washed in the PBS and the cellular debris and oocytes were filtered through a cell sieve (200 mesh). The filtered GCs and blood cells were collected into a 15 ml centrifuge tube and centrifuged at 1500 rpm for 5 min. Then, the supernatant was discarded and the mixed cells were washed with serum-free medium twice and cultured in a 12-well plate. After 24 hours, when the GCs had attached to the bottom of the culture plate, the remaining suspended blood cells were removed via medium replenishment.

For each experiment, six animals (mice and rats) were used for follicle isolation. Approximately ten intact and healthy follicles were isolated from each ovary for granulosa cell isolation and *in vitro* culture. GCs were cultured in DMEM/F12 (Gibco BRL/Invitrogen, Carlsbad, CA, USA) supplemented with 10% FBS and 1% penicillin–streptomycin. In some experiments, the cells were cultured in the presence of recombinant Activin A, FSH, BMP4 and BMP15 (R&D Systems, Minneapolis, MN, USA). The primary mouse or rat granulosa cells were passaged every 5–6 days depending on the cell density.

### Formation of atretic follicles

In mature mice and rats, a wave of mature oocytes will ovulate after PMSG treatment. Therefore, we selected approximately 23-day-old mice/rats at a premature stage without LH stimulation. Two days after PMSG injection, the oocytes become atretic but cannot be ovulated. Over time, an increasing number of follicles become atretic. We collected atretic follicles at 3, 4, 5 and 6 days post-treatment with PMSG.

### Fluorescence *in situ* hybridization

Normal ovaries from 23–26-day-old mice and rats (for miRNA localization in the normal ovary) and PMSG-injected mice and rats (for miRNA localization in the atretic follicles) were fixed in 4% paraformaldehyde for 20 minutes followed by overnight incubation in 30% sucrose. The ovaries were then embedded in Tissue Optimal Cutting Temperature Compound (Sakura, Horgen, Switzerland). miRCURY LNA miRNA detection probes for miR-10a and miR-10b were purchased from Exiqon (613307–310 and 613028–310, respectively; Vedbaek, Denmark). Fluorescence *in situ* hybridization was performed according to a standard protocol for microRNA FISH from Exiqon.

### miRNA mimic transfection

All of the cells were transfected with 20 nM of either miR-10a or miR-10b mimic (GenePharma) using the Lipofectamine RNAiMAX transfection reagent and Opti-MEM medium (Life Technologies) according to the manufacturer’s instructions. The control cells were transfected with 20 nM scrambled miRNA (GenePharma). Cells were incubated for 48 hours before being subjected to subsequent analysis unless otherwise mentioned.

### Proliferation assay

The cell proliferation rate was measured using a Cell-Counting Kit-8 (DOJINDO) according to the manufacturer’s protocol.

### Immunofluorescence

A total of 1 × 10^5^ cells were cultured on a cover glass in each well of a 12-well plate with 700 μl of medium. The cells were allowed to grow to the desired morphology and density before staining. To stain the cells, the cells were first washed once with PBS and fixed with 4% paraformaldehyde/4% sucrose in PBS at room temp, followed by permeabilization and DNA denaturation with 0.2% Triton X-100 in 4 M HCl. Next, the cells were washed with PBS and blocked in 80 μL BSA (3%). The cells were incubated with primary antibody BDNF (ab205067, Abcam, 1:100) in BSA (3%) overnight at 4 °C and then stained with Hoechst 33342 or DAPI. The glass slides were mounted with a cover slip before imaging.

### Apoptosis

Annexin V/propidium iodide (PI) staining was performed to detect apoptotic cells. Seventy-two hours after transfection, 5 × 10^5^ cells were collected and washed twice with ice-cold PBS. The cells were then stained using the Alexa Fluor^®^488 annexin V/Dead Cell Apoptosis Kit with Alexa^®^ Fluor 488 annexin V and PI for flow cytometry (Invitrogen, CA, USA) according to the manufacturer’s guidelines. Untreated cells served as a negative control for the double staining.

### Cell cycle

Cell cycle regulation was determined using a PI (Sigma, USA) staining assay.

### RNA-sequencing

Total RNA was quantified using a NanoDrop 2000 spectrophotometer (NanoDrop Technologies, Wilmington, DE). SVOG cells were collected 48 h after transfection with miR-10a/b mimics and/or negative control. To determine whole transcriptome expression, RNA-sequencing was performed with GROKEN Bioscience (China) according to a standard procedure.

### RT-qPCR

Total RNA was extracted with TRIzol reagent (Invitrogen, USA) according to a standard protocol. The concentration and quality of all the RNA samples were evaluated using a NanoDrop 2000 spectrophotometer (Thermo, USA), and the 260/280 and 260/230 values for all of the samples were above 1.8 and 1.9, respectively. Reverse transcription was performed with the MasterMix kit (Takara, Japan) and microRNA reverse transcription was performed with the TaqMan reverse transcription kit (Life Technologies, USA) following standard protocols. Quantitative PCR was performed using Universal SYBR Green Master mix (Applied Biosystems, USA), and microRNA qPCR was performed using TaqMan specific microRNA probes (Life Technologies) on a StepOnePlus real-time PCR system (Applied Biosystems). There were eight biological replicates per group. Additionally, eight technical replicates were used for each qPCR experiment. Gene expression was normalized to GAPDH, and microRNA expression was normalized to U6 unless otherwise stated. The primer list is included in the Supplementary File.

### Western-blot

Cells were lysed in SDS buffer. The protein concentration was measured by BCA assay kit (Thermo). Equal amounts of cell lysates were loaded, blotted onto a PVDF membrane, and probed with the following primary antibodies: anti-BDNF (ab205067, Abcam, 1:400), anti-GAPDH (D16H11, Cell Signaling, 1:2000) and anti-Actin (SC47778, Santa Cruz Biotechnology, 1:2000), which were used as loading controls. After incubation with the appropriate secondary antibodies, the signals were visualized by enhanced chemiluminescence (GE Systems, USA).

### Luciferase reporter assay

HEK293T cells grown in 24-well plates were transfected with 50 nM miR-10a and miR-10b mimic (GenePharma, China) and 100 ng of pmirGLO vector (Promega, USA) tagged with either a BDNF 3′ UTR that includes the miR-10 binding sites or the empty plasmid using Lipofectamine 2000 (Invitrogen, USA). The firefly and Renilla luciferase activities in the cell lysates were assayed with a Dual-Luciferase Reporter Assay System (Promega) at 48 h post-transfection.

### ShRNA and lentivirus system

A BDNF shRNA construct was designed using an online design program from MIT (http://sirna.wi.mit.edu/home.php). The 19-nucleotide hairpin-type shRNAs with a 9-nucleotide loop were cloned into the pSUPER-puro vector (OligoEngine, USA) according to manufacturers’ protocol. shRNA sequences are included in the Supplementary File. SVOG cells were transfected using Lipofectamine 2000 with the pSuper-puro shRNA constructs and selected for 4 days in media containing 2 μg/ml puromycin. The cells were collected and screened by western blot for target expression knockdown.

### Statistics

Data normality was tested with the D’Agostino & Person normality test and the Shapiro-Wilk Test. The error bars represent the standard error of the mean (SEM) for eight independent experiments. *, ** and *** indicate P < 0.05, P < 0.01 and P < 0.0001, respectively (Student’s t-test).

## Additional Information

**How to cite this article**: Jiajie, T. *et al*. Conserved miR-10 family represses proliferation and induces apoptosis in ovarian granulosa cells. *Sci. Rep.* **7**, 41304; doi: 10.1038/srep41304 (2017).

**Publisher's note:** Springer Nature remains neutral with regard to jurisdictional claims in published maps and institutional affiliations.

## Supplementary Material

Supplementary Information
