# Supplementary material for: Conserved miR-10 family represses proliferation and induces apoptosis in ovarian granulosa cells
Source: Sci Rep. 2017 Jan 23;7:41304. doi: 10.1038/srep41304 (PMC5256277; doi:10.1038/srep41304)
Supplement: Supplementary Information [file srep41304-s1.pdf]

Supplementary information:

Conserved miR-10 family represses proliferation and induces apoptosis in ovarian granulosa cells

Tu Jiajie[1,#], Yang Yanzhou[1,2], Albert Cheung Hoi-Hung[1], Chen Zi-Jiang[3], Chan Wai-Yee[1]*

[1] Chinese University of Hong Kong – Shandong University Joint Laboratory for Reproductive Genetics, School of Biomedical Sciences, Faculty of Medicine, the Chinese University of Hong Kong, Hong Kong SAR

[2] Key Laboratory of Fertility Preservation and Maintenance, Ministry of Education, Key Laboratory of Reproduction and Genetics in Ningxia, Department of Histology and Embryology, Ningxia Medical University, Yinchuan, Ningxia, 75004, P.R. China

[3] Center for Reproductive Medicine, Provincial Hospital Affiliated to Shandong University, Jinan, China; National Research Center for Assisted Reproductive Technology and Reproductive Genetics, China; The Key laboratory for Reproductive Endocrinology of Ministry of Education, China; Shandong Provincial Key Laboratory of Reproductive Medicine, Jinan, China

*To whom all correspondenc should be addressed at:
School of Biomedical Sciences, Room G03, Ground Floor, Lo Kwee-Seong Integrated Biomedical Sciences Building, The Chinese University of Hong Kong, Shatin, N.T., Hong Kong SAR, China.
Tel: +852-3943 1383
E-mail: chanwy@cuhk.edu.hk

[#] Current address: Institute of Clinical Pharmacology, Anhui Medical University, China

Supplementary figure 1. (A) The precursor sequences of miR-10 and miR-10b of human, mouse and rat. The red color highlights the mature sequences of miRNA and the green color indicates the difference of sequences among different species. (B) The amplification plot of qPCR shows the expression of miR-10a and miR-10b significantly increase after corresponding miRNA mimics transfection. (C) FISH results show the expression pattern of miR-10a and miR-10b in rat ovary. (D) Gene Ontology results of DEG in miR-10b overexpressed GCs. (E) Pathway analysis results of DEG in miR-10b overexpressed GCs. (F) The reverse expression patterns of BDNF and miR-10b in mouse ovary. (G) The western blot results show BDNF was repressed by shRNA in GCs. (H) The full-length western-blot results.

Supplementary table 1. All oligo sequences that included in this manuscript.

A

>hsa-miR-10a-5p
UACCCUGUAGAUCCGAAUUUGUG

>hsa-miR-10b-5p
UACCCUGUAGAACCGAAUUUGUG

>mmu-miR-10a-5p
UACCCUGUAGAUCCGAAUUUGUG

>mmu-miR-10b-5p
UACCCUGUAGAACCGAAUUUGUG

>Rho-miR-10a-5p
UACCCUGUAGAUCCGAAUUUGUG

>rno-miR-10b-5p
CCCUGUAGAACCGAAUUUGUGU

B

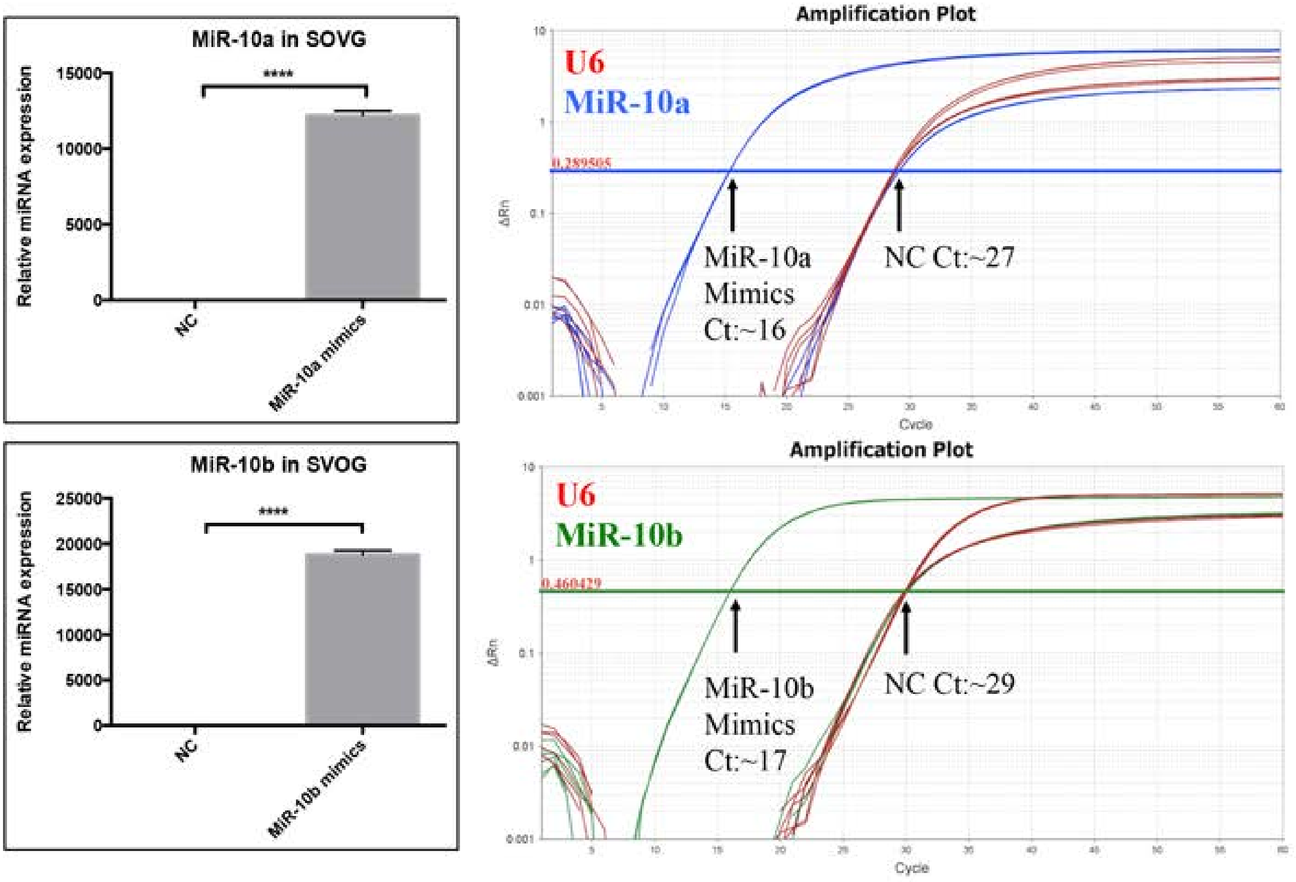

C

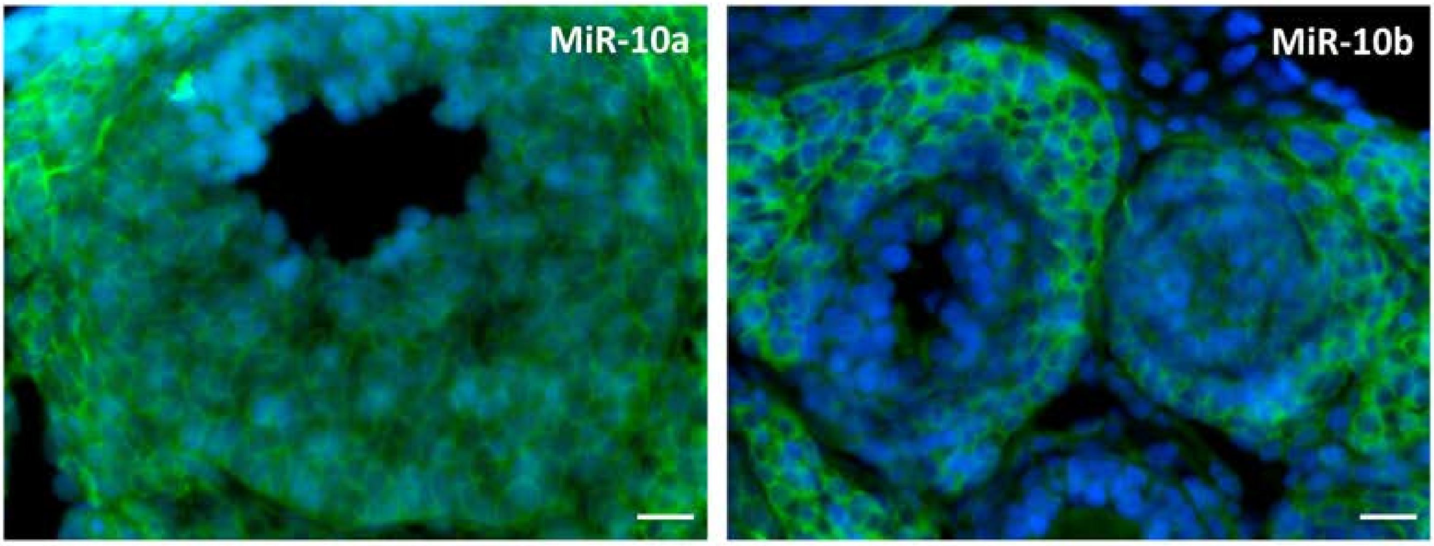

D

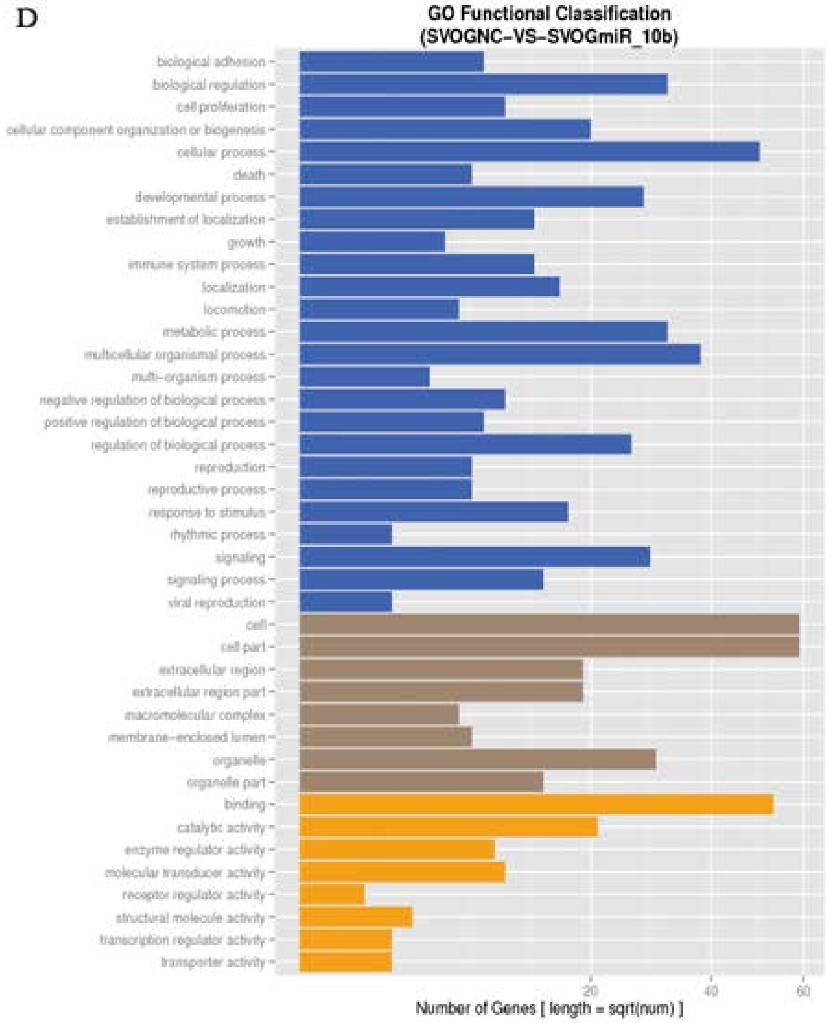

GO Functional Classification
(SVOGNC-VS-SVOGmiR_10b)
biological adhesion
biological regulation
cell proliferation
cellular component organization or biogenesis
cellular process
death
developmental process
establishment of localization
growth
immune system process
localization
locomotion
metabolic process
multicellular organismal process
multi-organism process
negative regulation of biological process
positive regulation of biological process
regulation of biological process
reproduction
reproductive process
response to stimulus
rhythmic process
signaling
signaling process
viral reproduction
cell
cell part
extracellular region
extracellular region part
macromolecular complex
membrane-enclosed lumen
organelle
organelle part
binding
catalytic activity
enzyme regulator activity
molecular transducer activity
receptor regulator activity
structural molecule activity
transcription regulator activity
transporter activity
biological_process
cellular_component
molecular_function
20
40
60
Number of Genes [length = sqrt(num)]

F

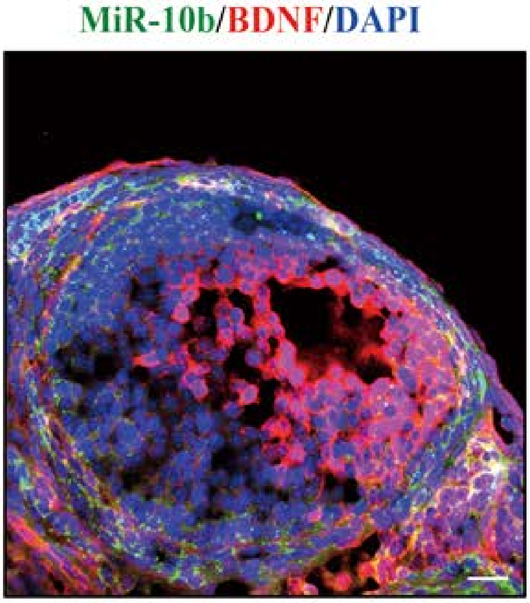

MiR-10b/BDNF/DAPI

G

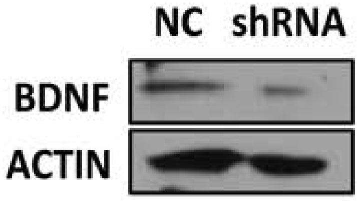

NC
shRNA
BDNF
ACTIN

E

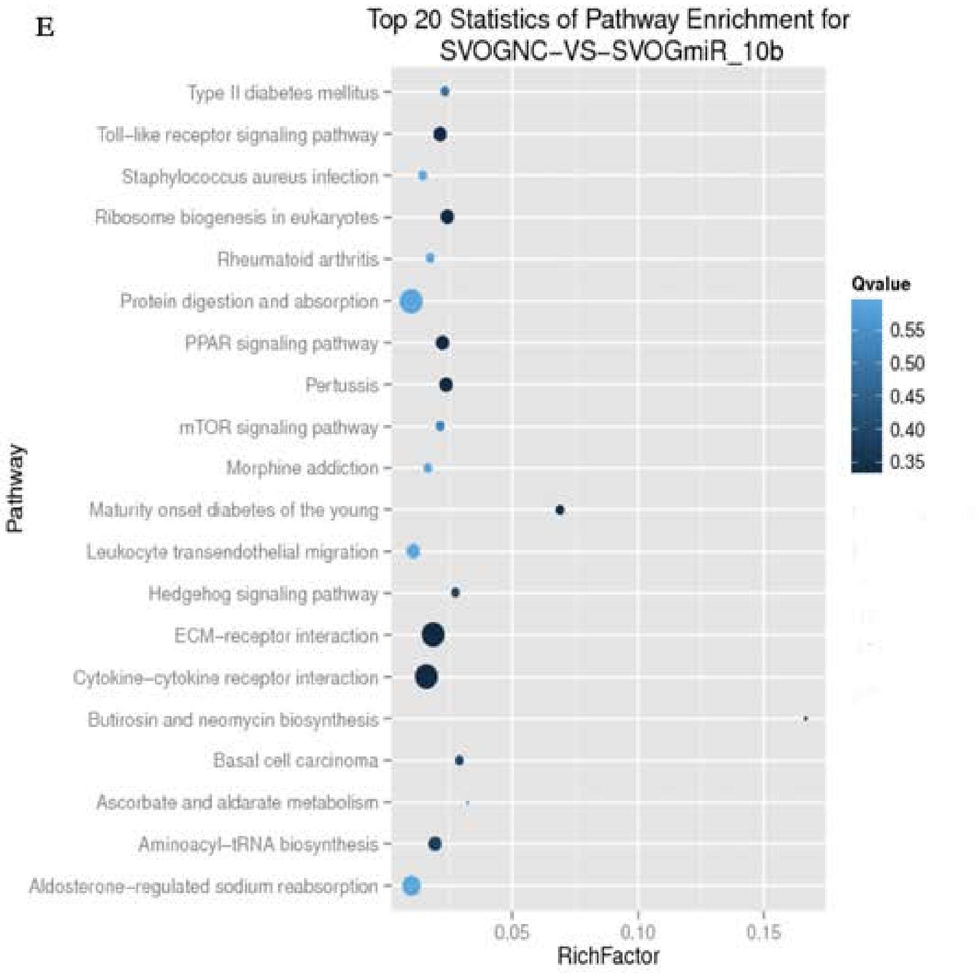

Top 20 Statistics of Pathway Enrichment for
SVOGNC-VS-SVOGmiR_10b
Type II diabetes mellitus
Toll-like receptor signaling pathway
Staphylococcus aureus infection
Ribosome biogenesis in eukaryotes
Rheumatoid arthritis
Protein digestion and absorption
PPAR signaling pathway
Pertussis
mTOR signaling pathway
Morphine addiction
Maturity onset diabetes of the young
Leukocyte transendothelial migration
Hedgehog signaling pathway
ECM-receptor interaction
Cytokine-cytokine receptor interaction
Butirosin and neomycin biosynthesis
Basal cell carcinoma
Ascorbate and aldarate metabolism
Aminoacyl-tRNA biosynthesis
Aldosterone-regulated sodium reabsorption
Pathway
0.05
0.10
0.15
RichFactor
Qvalue
0.55
0.50
0.45
0.40
0.35

H

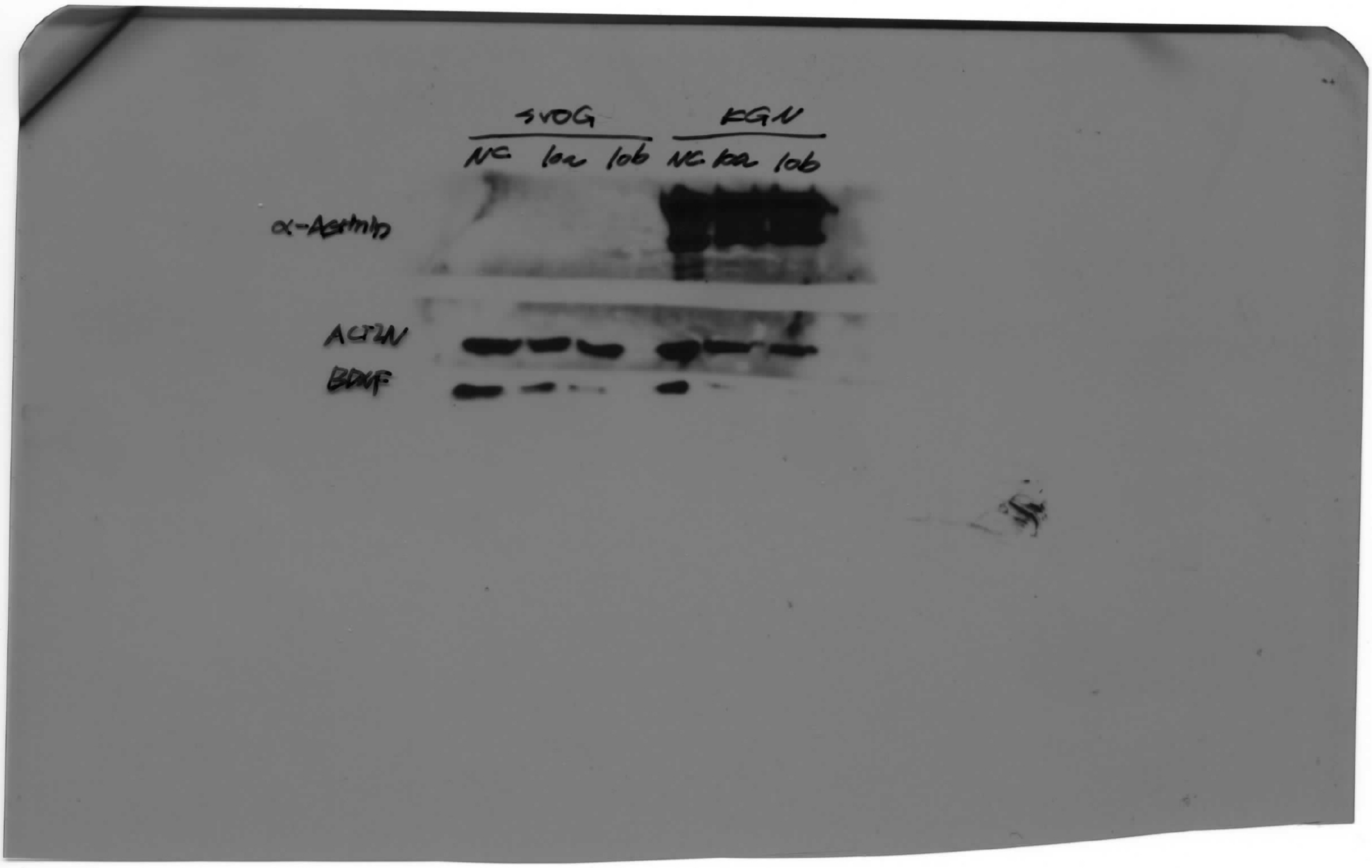

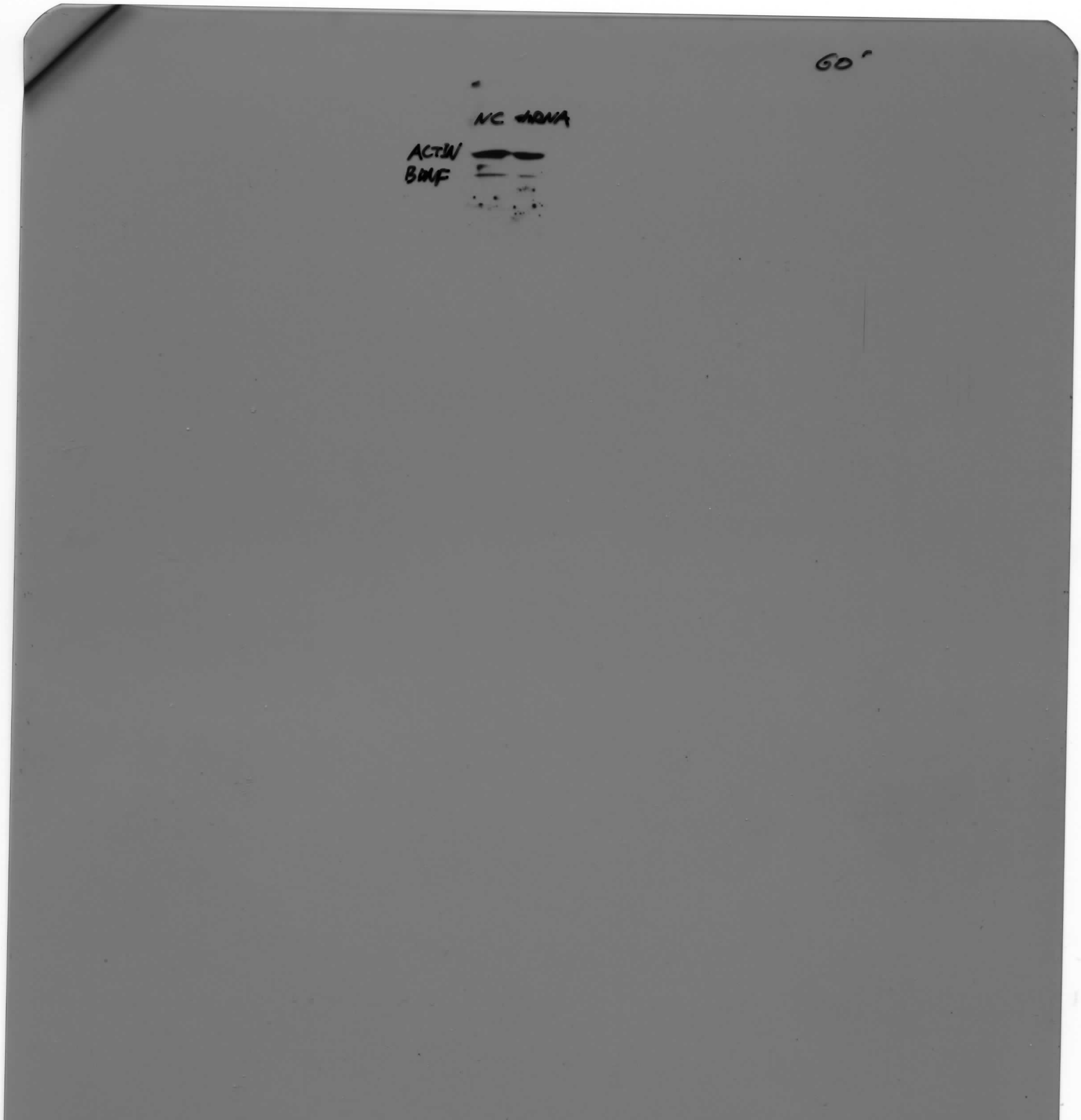

60'
NC
ACTIN
BMF

primer name	sequence
BDNF 1# shRNA F	GATCCCCGGCCAACTGAAGCAATACTTTCAAGAGAAGTATTGCTTCAGTTGGCCTTTTTA
BDNF 1# shRNA R	AGCTTAAAAAGGCCAACTGAAGCAATACTTCTCTTGAAAGTATTGCTTCAGTTGGCCGGG
BMP4 qPCR F	TGGGCTGGAATGACTGGATT
BMP4 qPCR R	TGGCATGGTTGGTTGAGTTG
AMH qPCR F	CAGCTCTATCACTGGGGAGG
AMH qPCR R	CAGACCCCTATCTCCTCTGC
BDNFqPCR F	CACCAAGACTTGAAGGTGCC
BDNFqPCR R	ATGAACAGACAGGATGGGCA
SMAD1 qPCR F	GTACTTCCTCCTGTGCTGGT
SMAD1 qPCR R	TGGAAAAGTGGCGTTGAGTG
SAMD3 qPCR F	GAGGGAGATGGAGAGGCTTC
SAMD3 qPCR R	CCTTCCGTCTCTTCCTGTGT
SAMD6 qPCR F	GATTCCCAGCAGCTCTTTGG
SAMD6 qPCR R	GATTTGCATGCACGAAAGGC
ACVR1 qPCR F	CCTAACCTCGCTCGATGACT
ACVR1 qPCR R	CTCCAGTCCCTACCTTTGCA
